# Molecular adsorbent recirculating system and hemostasis in patients at high risk of bleeding: an observational study

**DOI:** 10.1186/cc3985

**Published:** 2006-02-03

**Authors:** Peter Faybik, Andreas Bacher, Sibylle A Kozek-Langenecker, Heinz Steltzer, Claus Georg Krenn, Sandra Unger, Hubert Hetz

**Affiliations:** 1 Medical Doctor, Department of Anesthesiology and General Intensive Care, Medical University of Vienna, Austria; 2 Medical Technical Assistant, Department of Anesthesiology and General Intensive Care, Medical University of Vienna, Austria

## Abstract

**Introduction:**

Liver failure is associated with reduced synthesis of clotting factors, consumptive coagulopathy, and platelet dysfunction. The aim of the study was to evaluate the effects of liver support using a molecular adsorbent recirculating system (MARS) on the coagulation system in patients at high risk of bleeding.

**Methods:**

We studied 61 MARS treatments in 33 patients with acute liver failure (*n *= 15), acute-on-chronic liver failure (*n *= 8), sepsis (*n *= 5), liver graft dysfunction (*n *= 3), and cholestasis (*n *= 2). Standard coagulation tests, standard thromboelastography (TEG), and heparinase-modified and abciximab-fab-modified TEG were performed immediately before and 30 minutes after commencement of MARS, and after the end of MARS treatment. Prostaglandin I_2 _was administered extracorporeally to all patients; 17 patients additionally received unfractioned heparin.

**Results:**

Three moderate bleeding complications in three patients, requiring three to four units of packed red blood cells, were observed. All were sufficiently managed without interrupting MARS treatment. Although there was a significant decrease in platelet counts (median, 9 G/l; range, -40 to 145 G/l) and fibrinogen concentration (median, 15 mg/dl; range, -119 to 185 mg/dl) with a consecutive increase in thrombin time, the platelet function, as assessed by abciximab-fab-modified TEG, remained stable. MARS did not enhance fibrinolysis.

**Conclusion:**

MARS treatment appears to be well tolerated during marked coagulopathy due to liver failure. Although MARS leads to a further decrease in platelet count and fibrinogen concentration, platelet function, measured as the contribution of the platelets to the clot firmness in TEG, remains stable. According to TEG-based results, MARS does not enhance fibrinolysis.

## Introduction

The molecular adsorbent recirculating system (MARS) has been developed and successfully used in patients with liver failure to replace excretory liver function and detoxification. MARS is based on principles of albumin dialysis, and was shown to significantly improve hepatic encephalopathy, cerebral blood flow, renal function, and systemic hemodynamics [1-3]. It has further been shown that plasma concentrations of ammonia and many albumin-bound molecules, such as bilirubin, decreased during MARS therapy [4,5]. Nevertheless, improved outcome has been demonstrated in patiens with hepatorenal syndrome and acute-on-chronic liver failure [6,7].

Patients with liver failure exhibit major disturbances of hemostasis and are thus at a very high risk of bleeding. Decreased synthesis of clotting and inhibitory factors, decreased clearance of activated factors, quantitative and qualitative platelet defects, hyperfibrinolysis, and accelerated intravascular coagulation may all be present together in these patients [8]. Therefore, extracorporeal detoxification circuits, such as MARS, must be highly biocompatible and anticoagulatory measures to avoid clotting within the system must be tested for safety in such patients. On the basis of pathophysiological processes occurring after contact of blood with artificial surfaces, platelets are predominant in the genesis of extracorporeal thrombosis [9]. Therefore, attempts to run extracorporeal circulation without anticoagulation may result in frequent circuit clotting, except in severely thrombocytopenic patients [10], but with the risk of a further platelet loss [11]. Under these circumstances, extracorporeal inhibition of platelet function by prostaglandins combined with heparin has been shown to increase the biocompatibility of extracorporeal circulation [12,13].

The effects of extracorporeal support on hemostasis must be closely monitored in patients with liver failure to avoid major coagulation disbalances. The battery of traditional coagulation tests, which include prothrombin time, partial thromboplastin time, thrombin time, factor assays, and platelet function studies are based on isolated, static end points of standard laboratory tests [14]. They do not take into account the interaction of the clotting cascade and platelets in whole blood. Thromboelastography (TEG) allows assessment of haemostatic function, documenting the interaction of platelets with the protein coagulation cascade from the time of the initial platelet-fibrin interaction, through platelet aggregation, clot strengthening, and fibrin cross linkage to eventual clot lysis [15,16]. Moreover, different modified TEG methods allow the specific evaluation of platelet function and the effect of endogenous and/or exogenous heparinoids on plasmatic coagulation [17,18]. Using a TEG-guided algorithm, a reduction of blood and fluid requirements during liver transplantation has been demonstrated [19].

In the present study, we evaluated the effects of anticoagulation regimens on the coagulation system and bleeding events in patients with liver failure undergoing MARS therapy. Traditional coagulation tests, standard TEG, and modified TEG were used to comprehensively monitor coagulation.

## Materials and methods

### Patients

Data were retrospectively collected from intensive care unit records of all consecutive patients who underwent MARS treatment within two years in our ICU; these patients were treated for liver failure due to acute liver failure, acute-on-chronic liver failure, liver dysfunction after liver transplantation and sepsis. The local Ethics Committee waived the need for informed consent.

### Liver support

According to the method described by Mitzner and colleagues [7], MARS treatment was conducted through a conventional hemodialysis catheter placed in the jugulary or subclavian vein. Each treatment lasted from 8 to 24 hours. The extracorporeal blood circuit was driven using dialysis machine equipment (BM 25, Edwards Life Sciences, Saint-Prex, Switzerland). An albumin-impregnated, highly permeable dialyzer (MARS-Flux, Gambro, Lund, Sweden) was used, its membrane permitting the removal of protein bound toxins. A closed loop of 20% commercial serum albumin containing dialysate was used to guarantee the removal of the toxins from the dialysate side. The blood flow rates from the dialysis machine and the albumin dialysate circuit were equal at a rate of 150 to 200 ml/min over the albumin impregnated membrane. The albumin-enriched fluid was regenerated by perfusion through an anion exchanger column and an uncoated charcoal column, and low-flow dialyzer for dialysis.

### Anticoagulation

Prostaglandin I_2 _was continuously administered after the blood pump of the MARS cycle in all patients. In 17 patients, unfractioned heparin was additionally administered in the same way as prostaglandin I_2_. The decision to additionally administer unfractioned heparin was made by the intensivist on duty. The dose of unfractioned heparin was adjusted to maintain the activated clotting time between 120 and 140 seconds.

### Bleeding events

All bleeding events requiring transfusion greater than two units of packed red blood cells during, or within 24 hours from the start of MARS treatment were considered significant. All other bleeding events requiring two or less units of packed red blood cells, such as diffuse mucous bleeding, bleeding on the sites of central venous catheters, or gastrointestinal bleeding from the nasogastric tube, as well as blood product usage, were documented.

Fresh frozen plasma (FFP) was administered in patients who underwent invasive procedures, such as inserting of central venous catheter, and in all patients with moderate, or in some with continuous, mucous bleeding. Antithrombin (AT) was administered continuously when MARS coagulated in spite of anticoagulation with unfractioned heparin at low plasma AT levels.

### Laboratory analysis

Standard coagulation tests, including prothrombin time (PT; normal range 75% to 140%), activated partial thromboplastin time (aPTT; normal range 27 to 41 seconds) thrombin time (TT; normal range <17 seconds), fibrinogen concentration (normal range 180 to 390 mg/dl), AT (normal range 70% to 120%), and platelet count (normal range 150 to 350 G/l), were carried out as a daily routine before and after MARS treatment.

### Thromboelastography

TEG was performed as part of routine coagulation monitoring in our intensive care unit in patients with acute liver failure, during liver transplantation, and in patients with liver dysfunction and/or marked coagulopathy.

We performed standard TEG as well as heparinase-modified and abciximab-fab-modified TEG to enable detection of the underlying mechanism of coagulopathy in detail. Heparinase 1, an enzyme isolated from *Flavobacterium heparinum*, neutralizes heparin and heparin-like substances without affecting coagulation parameters in the TEG in the absence of heparin. The comparison of standard and heparinase-modified TEG permits quantification of heparin activity and allows the differentiation between heparin effects, coagulation factor deficiencies, and dilutional coagulopathy. Abciximab-fab is an antibody fragment against platelet glycoprotein IIb-IIIa. The interaction of platelets with fibrinogen is mediated via this receptor and is inhibited by the antibody fragment. Inhibition of platelet function with abciximab-fab permits quantitative assessment of the contribution of fibrinogen to clot strength. Modifications of the TEG with incubation of samples with abciximab-fab *in vitro *allows the differentiation between hypofibrinogenaemia and platelet dysfunction.

The first TEG was performed within one hour before commencement of MARS treatment (time point (TP) 1) to evaluate the coagulation status prior to extracorporeal circulation. The second TEG (TP2) was performed 30 minutes after the start of MARS treatment to evaluate the acute effects of extracorporeal circulation, and the third TEG (TP3) within one hour after the end of MARS treatment to exclude the residual effect of anti-haemostatic drugs used.

Blood samples for TEG were collected in polypropylene tubes containing buffered sodium citrate and assayed within ten minutes after withdrawal. For TEG testing, 1,000 μl of blood were activated for two minutes in 1% celite vials (Haemoscope, Morton Grove, IL, USA). The whole-blood TEG testing was performed in 360 μl of celite-activated blood. For heparinase-modified and abciximab-fab-modified TEG testing, 360 μl was incubated for two minutes with heparinase (IBEX Technologies, Montreal, Canada; specific activity, 109 IU/mg) in heparinase vials containing 4 IU/ml (Haemoscope). For abciximab-fab-modified TEG testing, 360 μl of heparinase-incubated blood was added to 5 μl abciximab-fab (ReoPro, Centocor, Leiden, Netherlands). All TEG preparations were recalcified with 20 μl 0.2 M CaCl_2 _and analyzed in a Thromboelastograph^® ^(Haemoscope) at 37°C.

**Figure 1:**
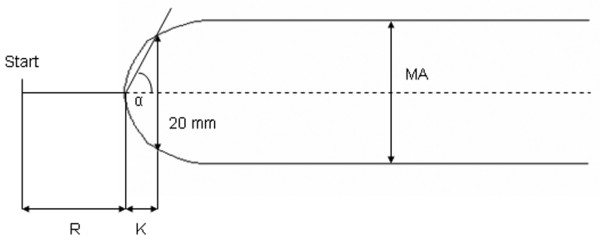
Normal thromboelastograph. α, angle alpha; K, coagulation time; MA, maximal amplitude; R, reaction time.

The TEG variables reaction time (R), clot formation time (K), angle alpha (α), maximum amplitude (MA), coagulation index (CI) and clot lysis after 30 minutes (CL30) were measured and documented (Figure 1). R (normal range 9 to 13 mm) is the time from sample placement in the TEG cup until the TEG trace amplitude reaches 2 mm. This represents the rate of initial fibrin formation and is functionally related to plasma clotting factors and circulating inhibitor activity. We also calculated the difference between standard R and heparinase modified R (standard R – heparinase modified R = R_HEP_). R_HEP _reflects the effects of endogenous and/or exogenous heparinoids on plasmatic coagulation. K (normal range 1 to 9 mm) is measured from R to the point where the amplitude of the tracing reaches 20 mm. It is the time taken to reach a standard clot firmness and is affected by the activity of the intrinsic clotting factors, fibrinogen and platelets. MA (normal range 45 to 53 mm) is the maximal amplitude on the TEG trace. It reflects the strength of the clot and is a direct result of the function of platelets and plasma factors and their interaction. We also calculated the difference between standard MA and abciximab-fab modified MA (standard MA – abciximab-fab MA = MA_PLT_). MA_PLT _reflects the contribution of platelets to the clot firmness. Angle α (normal range 55 to 62 mm) is the angle formed by the slope of the TEG tracing from the R to the K value. It represents the rate of clot growth and describes the polymerization of the structural elements involved in clotting. Clot growth is a function of platelets and plasma components residing on the platelet surfaces. CL30 (normal range 100%) is a measure of clot retraction or lysis. CI (normal range -3 to 3 mm) is an overall indicator of coagulation and indicates normal, hypo- or hypercoagulable state.

### Statistical analysis

Data are presented as median and 25th to 75th percentile unless indicated otherwise. Normal distribution of samples was tested with the Kolmogorov-Smirnov test. Serial results were compared by Friedman repeated-measures analysis of variance on ranks. The non-parametric Wilcoxon rank test was used to compare pre- and post-MARS variables, and the Mann Whitney *U *test to test the differences between the patients with and without additional heparin for anticoagulation or fresh frozen plasma administration. *P *< 0.05 was considered statistically significant. All statistical analyses were performed with the software Statview^® ^5.0 for Windows (SAS Institute Inc., Cary, NC, USA).

## Results

We have studied 61 MARS treatments performed in 33 consecutive patients (24 men, 9 women) at high risk of bleeding. Of these, 21 patients (63%) survived more than three months and 12 died. The indications for MARS support were acute liver failure (*n *= 15), acute-on-chronic liver failure (*n *= 8), liver dysfunction after liver transplantation (*n *= 3), septic liver dysfunction (*n *= 5) and cholestasis with pruritus (*n *= 2). Of the patients with acute liver failure, 7 out of 15 were successfully bridged toward liver transplantation and underwent uneventful liver transplantation. Patient characteristics, baseline laboratory values, the sepsis-related organ failure assessment (SOFA) score and the model of end-stage liver disease (MELD) score are shown in Table 1. All patients were anticoagulated with 3 to 5 ng/kg/minute PGI_2 _during the MARS treatment; 17 (51%) patients additionally received 100 to 600 IE unfractioned heparin in 34 (55%) MARS treatments. AT was continuously administered in 6 (18%) patients during 11 (18%) MARS treatments. And 15 patients (45%) received FFP (median, 4 units; range, 2 to 8 units) in 37 (60%) MARS treatments.

The MARS treatment lasted for 16 (9 to 19.2) hours. Treatment times in patients receiving unfractioned heparin in addition to PGI_2 _were not significantly different compared to those with PGI_2 _alone; 16 (10 to 19) hours versus 16 (8 to 19.7) hours, respectively (*p *= 0.76). Furthermore, application of FFP had no significant effect on duration of MARS treatment (treatment time 16 (9 to 18.5) hours without FFP administration versus 17.5 (12 to 20) hours with FFP administration (*p *= 0.33). Twelve (19%) MARS circuits clotted during the treatment. Consequently, there was a significantly shorter treatment time in MARS circuits that clotted than in those that did not; 8 (7 to 13) hours versus 17 (12.2 to 20) hours, respectively (*p *= 0.0007).

Three moderate bleeding complications, defined as significant bleeding events, occurred in three (9%) consecutive patients requiring more than two units (range three to four units) of packed red blood cells, one platelet concentrate and six to eight FFPs. The first was attributable to the deterioration of the clinical course of end-stage liver disease after hepatic surgery in cirrhosis and the patient was not administered heparin during MARS treatment. The other two moderate bleeding events were most probably related to the effect of heparin. All were sufficiently managed without interrupting MARS. Altogether, during or within 24 hours from the start of MARS treatment, a median of two units (range 1 to 4 units) of packed red blood cells were administered in 17 (51%) patients during 27 (44%) MARS treatments. There were five patients (15%) who presented with either mucous bleeding and/or bleeding from the insertion site of the central venous catheters already before the start of 13 (21%) MARS treatments. Two patients (6%) started to bleed from the insertion site of the central venous catheters for the first time during MARS treatment. None of the patients with minor bleeding received unfractioned heparin. However, none of these superficial bleeding events led to discontinuation of the extracorporeal treatment. The other patients presented with anemia either due to critical illness and/or frequent blood sampling over their stay in the intensive care unit. There was no significant difference in the requirement for packed red blood cells between patients with renal failure and those with normal renal function (*p *= 0.89).

### Effects of MARS on standard coagulation tests

There were no significant changes in PT, PTT and AT during MARS treatment in all patients (Table 2). The TT increased significantly. Fibrinogen concentration and platelet count decreased significantly after MARS treatment. The median decreases in fibrinogen concentration and platelet count per MARS treatment were 15 mg/dl (-11 to 45 mg/dl) and 9 G/l (0 to 20 G/l), respectively.

There was a significantly higher PTT in patients not administered unfractioned heparin before commencement of MARS (median 64 s versus 53 s, *p *= 0.02), but this significance disappeared after the MARS treatment.

No significant difference in plasma AT levels was detected before (*p *= 0.9), although there was a significant difference in plasma AT levels after, MARS treatments between patients who were administered AT and those who were not (*p *= 0.01). In accordance with these results, plasma AT levels increased significantly in patients who received AT (*p *= 0.04) and decreased significantly in those who did not (*p *= 0.003).

FFP administration during MARS treatment had no significant effects on changes of PT, PTT, TT, fibrinogen concentration and AT. In patients who were not administered FFP during MARS treatment, TT increased from 16.1 to 17.3 s (*p *= 0.01), and fibrinogen concentration decreased from 150 to 142 mg/dl (*p *= 0.002); PT, PTT and AT showed no significant changes.

### Effects of MARS on standard TEG

The effects of MARS support on TEG variables are summarized in Tables 3 and 4. Both MA and angle α differed significantly between TP1 and TP3 (*p *< 0.05) and TP2 and TP3 (*p *< 0.05) but not between TP1 and TP2 (*p *> 0.05). This means that the changes occurred later than 30 minutes after the beginning of MARS treatment.

### Effect of MARS on platelet function in abciximab-fab-modified TEG

MA_PLT _did not change significantly in all patients (*p *= 0.3). MA_PLT _did not change significantly in patients who received unfractioned heparin in addition to prostaglandin I_2 _for anticoagulation. We did not measure a significant difference in MA_PLT _between patients who were administered 3 or 5 ng/kg/minute prostaglandin I_2_, and observed no effect of prostaglandin I_2 _on MA_PLT _between TP1 and TP2 at all.

### Effect of MARS on plasmatic coagulation in heparinase-modified TEG

The effect of MARS support on R in heparinase-modified TEG in all patients as well as in patients who were administered FFP are summarized in Tables 3 and 4. *Post hoc *analysis of R_HEP _in patients without FFP administration revealed a significant increase from TP1 to TP2 (*p *= 0.04) and from TP1 to TP 3 (*p *= 0.003), but not between TP2 and TP3 (*p *= 0.86).

R_HEP _increased significantly (*p* = 0.04) in patients who received unfractioned heparin additionally to prostaglandin I_2_. In those patients who received prostaglandin I_2 _solely, R_HEP _did not change significantly (*p *= 0.67). *Post hoc *analysis of R_HEP _in patients anticoagulated with both PGI_2 _and unfractioned heparin revealed a significant difference between TP1 and TP2 (*p *= 0.009) and TP1 and TP3 (*p *= 0.04), but not between TP2 and TP3 (*p *= 0.36). This documents the effect of exogenous heparin on plasmatic coagulation. Although there was a greater effect of endogenous heparinoids at TP1 in patients who were not administered unfractioned heparin than in those who were, this difference reached no statistical significance (*p *= 0.2).

### Effect of fresh frozen plasma administration on coagulation tests and TEG variables

FFP was administered in 37 MARS treatments in 15 patients. In all patients, the FFP was administered later than 30 minutes (TP2) after the start of the MARS treatment. To exclude this bias and to study the MARS effects solely, we evaluated these patients separately (Table 4).

Considering only the patients that were not administered FFP, there was, among the standard battery of coagulation tests, only a slight but significant decrease in fibrinogen, leading to an increase in TT. Although these changes reached statistical significance, they are too low to lead to clinical deterioration of coagulation. In standard TEG, α and MA decreased significantly. All these changes occurred later than 30 minutes after the start of MARS; therefore, no acute deterioration of coagulation due to contact of blood with the surface of the extracorporeal circuit occurred. Bearing in mind the decrease of fibrinogen and no change in platelet function measured by modified TEG in our patients, all these slight but significant changes of standard TEG variables may result from the decrease in fibrinogen concentration.

If we focus on only the patients who received FFP during the MARS treatment, nearly no, or at least no significant, effects were seen with the standard coagulation tests. Among the TEG variables, K and MA worsened slightly but significantly after the end of MARS treatment (TP1 versus TP3, *p *< 0.05).

## Discussion

Our results provide evidence that MARS support is well tolerated in patients with marked coagulopathy and low platelet count. Although nearly all treated patients exhibited major coagulation abnormalities in both standard coagulation tests and TEGs, no serious or uncontrollable bleeding events attributable solely to the MARS therapy were observed. This clinical observation is supported by the TEG parameter clot lysis, which did not change significantly during MARS treatment. The enhanced fibrinolysis and low grade disseminated intravascular coagulation are recognized as common features in advanced liver disease [20,21]. According to our TEG-based results, however, MARS treatment did not enhance fibrinolysis.

The major result in coagulation tests was a significant decrease in platelet count during MARS therapy. Platelet loss has also been reported by other groups using MARS treatment [4,6,16]. In our study population, however, the median platelet loss of 9 G/l was much lower than the median decrease of 49 G/l seen with cuprophane charcoal-based detoxification [22]. This is probably due to avoiding direct contact between whole blood and the charcoal and anion exchanger columns.

Clinically important, platelet loss was not accompanied by deterioration of platelet function. According to the study by Doria and colleagues [16], who found a significant decrease in platelet count and the standard MA, MARS induces coagulopathy through a platelet-mediated mechanism caused either by a mechanical destruction in the filters and lines or by an immune-mediated process. We further performed abciximab-fab-modified TEG and it revealed that the MA_PLT _remains unchanged. Therefore, the change of MA in the standard TEG is due to a decrease in fibrinogen concentration and not due to deterioration of platelet function. Indeed, the fibrinogen concentration decreased significantly in all patients, leading to prolonged TTs.

Decrease in fibrinogen concentration and prolongation of TT was not observed in the subgroub of patients recieving FFP. The amount of FFP administered appeared to be sufficient to substitute for the low coagulation status and did not enhance filter clotting, as indicated by treatment time.

This is the first study focusing on effects of anticoagulation in patients at high risk of bleeding undergoing MARS treatment. The previous study by Doria and colleagues [16], which also applied TEG in addition to standard coagulation tests, was performed without any anticoagulation. The use of anticoagulation in patients at high risk of bleeding is still a matter of discussion. Different approaches have been used so far, including heparin flush, systemic heparin, short acting prostaglandins, citrate, or no anticoagulation at all [10,23-25]. Based on the pathophysiological process of platelet activation by contact with a layer of plasma proteins on the artificial surface and consecutive release of granular contents leading to initiation of thrombi formation [11,26], we used short acting prostaglandin I_2 _to inhibit platelet activation. Recently, prostaglandin I_2 _was shown to reversibly inhibit platelet function by diminishing the expression of platelet fibrinogen receptor and P-selectin, and to reduce heterotypic platelet-leukocyte aggregation during clinical hemofiltration [27]. Furthermore, supplementary prostaglandin I_2 _to unfractioned heparin enhanced hemofilter duration in continuous venovenous hemofiltration [13]. Surprisingly, prostaglandin I_2 _administration in our study was not accompanied by any changes in the coagulation tests. This may be due to either a lack of diagnostic accuracy of TEG [28], or a lack of prostaglandin I_2 _activity in systemic circulation due to its short half-life of three to five minutes. Indeed, we performed TEG on patients' blood drawn from an arterial line and not in the blood from the extracorporeal circulation, where PGI_2 _was administered. It was shown in another study examining anticoagulation with prostaglandin I_2 _and heparin during venovenous hemofiltration that there are significantly higher concentrations of 6-ketoprostaglandin F_1α_, the degradation product of prostaglandin I_2_, in extracorporeal than in systemic blood during extracorporeal administration of prostaglandin I_2 _[13].

In all our patients, we detected increased heparin-like effects on coagulation parameters before MARS treatment, indicated by the significant difference between standard and heparinase-modified TEG. This reflects higher levels of endogenous heparinoids resulting from decreased elimination by the failing liver [17]. Patients who additionally received unfractioned heparin showed further significant increases in R_HEP _and aPTT, reflecting the effect of exogenous heparin administration and not the effect of MARS treatment on plasmatic coagulation.

Patients with liver failure can present as both hypocoagulant and hypercoagulant [8], as seen in the broad range of the coagulation index from -22 to 20 (normal range -3 to +3) in our patients. In one of the two patients with moderate bleeding complications who received heparin in addition to prostaglandin I_2_, heparin at 200 IE/h was administered during three MARS treatments because high transmembraneous pressure was documented and small clots were suspected in the filters of the extracorporeal lines. The coagulation index of this patient ranged from -1.52 to 3.37, within normal to hypercoagulable TEG values of overall coagulation, in spite of marked coagulopathy indicated by standard coagulation tests. In the other patient, two MARS treatments performed with prostaglandin I_2 _alone had to be terminated after two hours due to clotting of the venous port. This phenomenon had been detected in TEG as a hypercoagulable state in spite of abnormal coagulation tests. Additional unfractioned heparin and AT administration prolonged the circulation lifespan in the third treatment, but led to consecutive bleeding in the fourth. The activating clotting time measured in 30 minute intervals remained stable between 120 and 140 seconds and appeared to be prolonged only when the bleeding occurred. These patients clearly represent the fluid and dynamic changes of coagulation in patients with liver failure and provide evidence for the importance of point-of-care monitoring. Furthermore, these results also indicate that unfractioned heparin is not the ideal anticoagulant in this situation.

In contrast to the traditional coagulation tests, which are based on isolated, static end points of standard laboratory tests, TEG takes into account the interaction between the clotting cascade and platelets in whole blood. We found standard and modified TEG very useful, especially in patients who presented with marked coagulopathy in spite of a hypercoagulable state indicated by TEG. Based on these experiences, standard and modified TEG became an important tool for monitoring coagulation during MARS treatment in our intensive care unit.

This study has several limitations. Firstly, it is a retrospective observational study and thus lacks the structure of a prospective study conducted according to a specific protocol. Secondly, there are many groups of patients suffering from different etiologies leading to liver failure. Although the common point of each of the groups is the fact that all of them have coagulation problems, some doubts may be raised because the results are not significant. Thus, only a study with larger populations of different etiologies leading to liver failure can address this issue.

## Conclusion

MARS treatment appears to be well tolerated in thrombocytopenic patients with marked coagulopathy due to liver failure. Although this form of liver support leads to a further decrease in platelet count and fibrinogen, platelet function, measured as the contribution of the platelets to clot stiffness in TEG, remains stable. According to TEG-based results, MARS treatment does not enhance fibrinolysis. TEG provides a useful additional tool to monitor coagulation during MARS treatment.

## Key messages

• 	Treatment with Molecular Adsorbent Recirculating System is well tolerated in thrombocytopenic patients with marked coagulopathy.

• 	In spite of a slight decrease in platelet count and fibrinogen, platelet function, assessed as the contribution of the platelets to clot stiffness in modified thromboelstography, is not affected.

## Abbreviations

α = angle alpha; aPTT = activated partial thromboplastin time; AT = antithrombin; CI = coagulation index; CL30 = clot lysis after 30 minutes; FFP = fresh frozen plasma; K = clot formation time; MA = maximum amplitude; MARS = molecular adsorbent recirculating system; MELD = model of end-stage liver disease; PT = prothrombin time; R = reaction time; SOFA = sepsis related organ failure assessment; TEG = thromboelastography; TP = time point; TT = thrombin time.

## Competing interests

This study was supported by a grant obtained from Biotest Pharmazeutika GmbH, Vienna, Austria. The authors have no financial interests relevant to the results of this research, nor are there any other circumstances that could potentially provoke a conflict of interest.

## Authors' contributions

PF and HH conceived the study, participated in its design and coordination, performed the statistical analysis and helped to draft the manuscript. SU carried out the thromboelastographs and participated in its design. AB and SK participated in the study design and helped to draft the manuscript. CK and HS participated in the sequence alignment and drafted the manuscript. All authors read and approved the final manuscript.

## Figures and Tables

**Table 1:** Baseline demographic data and laboratory variables before first molecular adsorbent recirculating system treatment

	Median	Range
Weight (kg)	74.5	30–105
BSA (kg/m^2^)	1.9	1.5–2.3
Age (years)	51	10–65
SOFA	13.5	0–22
MELD	28.7	5–44
Bilirubin (mg/dl)	11.3	0.5–42
Creatinine (mg/dl)	1.46	0.4–4.9
AST (U/l)	294	24–18,750
ALT (U/l)	436	20–6,410
Albumin (mg/dl)	24.1	10–48
PT (%)	33	5–94
Hematocrit (%)	29.5	22–45

ALT, alanine aminotransferase; AST, aspartate aminotransferase; BSA, body surface area; MELD, model of end-stage liver disease; PT, prothrombin time; SOFA, sepsis related organ failure assessment.

**Table 2:** Standard coagulation tests before and after molecular adsorbent recirculating system treatment (pooled data)

Parameter	Before MARS treatment		After MARS treatment		*p*
	Median (interquartile range)	Range	Median (interquartile range)	Range	

PT (%)	28 (16–40.2)	5–129	27 (17.5–46.2)	5–92	0.93
aPTT (s)	57.6 (49.5–68.2)	32–103	54 (46.9–70.5)	33–121	0.94
TT (s)	16.7 (14.8–20.1)	10–120	17.6 (15.1–21.1)	11.4–60	0.02
Fibrinogen (mg/dl)	145 (91.7–312)	42–1,120	142 (74.7–319)	13–1,020	0.006
AT (%)	43 (27–61)	12–127	42 (26.7–59.2)	8–120	0.14
Platelets (G/l)	60.5 (28.5–85.5)	8–352	44 (23–77.5)	6–254	<0.0001
Hematocrit (%)	30 (27–33)	22–45	30 (27–32.5)	19–40	0.95

AT, antithrombin; aPTT, activated partial thromboplastin time; PT, prothrombin time; TT, thrombin time.

**Table 3:** Standard and modified thromboelastography in all studied patients (pooled data)

	TP1	TP2	TP3	*p*
Reaction time (mm)	16.7 (13.3–21.6)	17.5 (13.8–21.1)	17.4 (12.9–21.3)	0.84
Coagulation time (mm)	12 (7.3–25.1)	13.7 (7.5–33.2)	15.0 (5.85–33.0)	0.07
Maximal amplitude (mm)	38.5 (26.8–48.8)	35.5 (25.1–50.2)	34.0 (22.7–48.2)	0.0003
Angle alpha (degree)	35 (24.5–51.1)	42 (23.7–54.8)	30.0 (17.8–53.5)	0.002
Clot lysis (percent)	100 (98.7–100)	100 (99.1–100)	100 (98.4–100)	0.68
Coagulation index	-0.68 (-6.3–3.9)	-1.0 (-6.3–3.6)	-1.5 (-7.7–4.6)	0.81
R_HEP_(mm)	0.7 (-0.5–2.6)	2.9 (0.125–5)	1.4 (0.2–4.1)	0.07
MA_PLT _(mm)	24 (12.5–31.1)	20 (13.6–28.6)	19 (8.1–29.5)	0.3

MA_PLT_, difference between standard and abximimab-fab-modified maximal amplitude (MA) reflecting solely the platelet function. R_HEP_, difference between standard and heparinase modified reaction time (R), reflecting the effects of endogenous/exogenous heparinoids on plasmatic coagulation. TP, time point.

**Table 4:** Standard and modified thromboelastography with and without fresh frozen plasma administration (pooled data)

	TP1	TP2	TP3	*p*
Patients with FFP administration				
Reaction time (mm)	19.4 (13.8–25.6)	20 (15.9–23.9)	17.4 (15.1–23)	0.46
Coagulation time (mm)	16 (9.1–37.8)	25.5 (10.2–45.7)	25.1 (13–33.9)	0.02
Maximal amplitude (mm)	34.2 (23.2–42.2)	29 (22.5–43)	29.7 (20.5–40.5)	0.015
Angle alpha (degree)	30.2 (17–51.2)	34.2 (18.5–45.7)	21 (17.5–37)	0.06
Clot lysis	100 (100-100)	100 (100-100)	100 (100-100)	0.84
Coagulation index (percent)	-2.2 (-14.4–2.5)	-5 (-11.5–1.9)	-4.7 (-8.6–1.1)	0.9
R_HEP _(mm)	1 (-0.7–2.2)	3 (-0.1–6.8)	0.7 (-0.6–4.1)	0.64
MA_PLT _(mm)	20 (12–30.3)	21.5 (15–31)	24 (13.8–32.2)	0.22
				
Patients without FFP administration				
Reaction time (mm)	15.9 (13–19.4)	16.8 (13–19.3)	16.4 (10.9–20.5)	0.71
Coagulation time (mm)	11.3 (7–19.7)	9.4 (6.3–21)	14.2 (5–32)	0.6
Maximal amplitude (mm)	40 (32–52)	37.5 (28.3–52.6)	36.5 (24.8–53.3)	0.006
Angle alpha (degree)	35.5 (28.5–51)	47.5 (30–57)	35.5 (21.2–57.7)	0.028
Clot lysis (percent)	100 (98.6–100)	100 (98.6–100)	100 (97.8–100)	0.77
Coagulation index	0.38 (-3.4–4.2)	-0.1 (-2.3–3.8)	0.3 (-7.7–4.8)	0.73
R_HEP _(mm)	0.7 (-0.4–2.8)	2.9 (0.3–4.7)	1.9 (0.7–4)	0.03
MA_PLT _(mm)	24.4 (12.5–31.2)	20 (8.5–27.5)	13.5 (6.2–27.5)	0.95

MA_PLT_, difference between standard and abximimab-fab-modified maximal amplitude (MA) reflecting solely the platelet function. R_HEP_, difference between standard and heparinase modified reaction time (R) reflecting the effects of endo/exogenous heparinoids on plasmatic coagulation. FFP, fresh frozen plasma; TP, time point
